# Editorial: Optical Molecular Imaging in Cancer Research

**DOI:** 10.3389/fonc.2022.870583

**Published:** 2022-03-28

**Authors:** Guanglei Zhang, Xueli Chen, Shouju Wang, Jiao Li, Xu Cao

**Affiliations:** ^1^ Beijing Advanced Innovation Center for Biomedical Engineering, School of Biological Science and Medical Engineering, Beihang University, Beijing, China; ^2^ Xi’an Key Laboratory of Intelligent Sensing and Regulation of trans-Scale Life Information, School of Life Science and Technology, Xidian University, Xi’an, China; ^3^ Department of Radiology, The First Affiliated Hospital of Nanjing Medical University, Nanjing, China; ^4^ School of Precision Instruments and Optoelectronics Engineering, Tianjin University, Tianjin, China; ^5^ Thayer School of Engineering, Dartmouth College, Hanover, NH, United States

**Keywords:** optical molecular imaging, cancer imaging, biological applications, imaging systems, imaging methods

## Preface

Optical molecular imaging (OMI) is an emerging technology capable of qualitatively and quantitatively studying life processes at the cellular or molecular level (1). Compared with traditional medical imaging technologies, it can detect the occurrence of diseases in advance without obvious morphological changes in the early stages of the pathological process, and can perform real-time, non-invasive continuous dynamic monitoring *in vivo* (2).

OMI technology has developed rapidly in recent years owing to its high sensitivity, strong specificity, and fast imaging speed. OMI technologies mainly include bioluminescence imaging (BLI), fluorescence molecular imaging (FMI), x-ray luminescence imaging (XLI), Cerenkov luminescence imaging (CLI), and photoacoustic imaging (PAI), etc, which can monitor the biological and pathological activities at the cellular or molecular level. Since the conventional OMI technology can only provide two-dimensional (2D) images, the corresponding three-dimensional (3D) imaging technologies, including bioluminescence tomography (BLT) (3), fluorescence molecular tomography (FMT) (4), x-ray luminescence tomography (XLT) (5), Cerenkov luminescence tomography (CLT) (6), photoacoustic tomography (PAT) (7), diffusion optical tomography (DOT) (8), etc, are also intensively explored and developed to provide 3D quantitative information.

This Research Topic aims to collect multiple applications of the 2D and 3D OMI technologies used to carry out cancer studies, including studies of novel imaging systems, imaging methods, probes, drugs, and biological applications in the field of cancer research.

## Biomedical and Clinical Research on Cancers

### Brain Cancer

Lukina et al. utilized fluorescence lifetime imaging (FLIM) of endogenous fluorophores related to the metabolism of the glioma to develop a rapid and sensitive assay for intraoperative diagnostics of glioma and identification of the optical markers essential for differentiation between tumors and healthy brain tissues. They found that fluorescence lifetime parameters of the glioma provided a background for differentiation between the tumors and brain tissue. Experiments using tumors from both rats and patients demonstrated substantial differences between the malignant tumors and normal tissues.

### Breast Cancer

Li et al. prepared a novel ultrasound contrast agent VEGFR2-targeting iron-doped silica (SiO2) hollow nanoparticles (VEGFR2-PEG-HSNs-Fe NPs) and applied it in microwave ablation for breast cancer to investigate its value in the evaluation of effectiveness after tumor ablation. The subcutaneous xenograft tumor was established to simulate the microenvironment of mouse breast cancer. After the xenograft tumor was treated with microwave ablation, the extent of perfusion defect was evaluated by injecting VEGFR2-PEG-HSNs-Fe NPs, and an enhanced ultrasound signal was detected in the tumor. Experiments showed that the nano-targeted VEGFR2-PEG-HSNs-Fe NPs had good biosafety and ability for specific imaging, which had the potential to evaluate the efficacy of tumor ablation.

Li et al. designed new types of nanocarriers, the cascade release near-infrared imaging (NIFI) and thermal-chemo combination nanoparticles (CNC NPs), which can release drugs through the cascade of ultrasound triggering and pH responding to achieve synchronous tumor accumulation, monitoring, and the synergistic treatment of two functional molecules. The NPs can effectively perform cascade drug release through ultrasound triggering and pH responding. The CNC NPs have good *in vivo* biological safety and excellent fluorescence imaging, drug delivery, and therapeutic abilities in triple-negative breast cancer models.

### Gastric Cancer

Yin et al. prepared 68Ga-DOTA-KEK-(GX1)2 and applied it to PET and Cerenkov imaging of gastric cancer. Its tumor-targeting ability was determined by nano PET/CT and Cerenkov imaging, standardized uptake value, signal-to-background ratio (SBR) quantification, and a bio-distribution study in tumor-bearing nude mice. Experiments showed that GX1 was modified successfully, and the *in vivo* and *in vitro* properties of the GX1 dimer were significantly better than those of GX1. The imaging probe 68Ga-DOTA-KEK-(GX1)2 is a potential candidate probe for PET and Cerenkov diagnosis of gastric cancer.

### Liver Cancer

Yan et al. performed a phage-displayed bio-panning to identify a specific binding peptide targeting Glypican-3 (GPC-3), which could be used as a biomarker in hepatocellular carcinoma (HCC). In this bio-distribution study, a higher accumulation of F3 peptides was observed in HepG-2 tumors compared to PC-3 tumors in xenograft models. Furthermore, the F3 peptide tracer enabled the specific detection of tumors in HCC tumor models with PET imaging after labeling with radioactive 68Ga. This cyclic peptide targeting GPC-3 may be an alternative to serve as an imaging probe or a targeting domain in the drug conjugate.

Zhou et al. utilized IR780 as the near-infrared fluorescence imaging, photoacoustic imaging, and photothermal therapy (PTT) agent, and utilized paclitaxel (PTX), which is a broad-spectrum chemotherapy drug, together to build the NIF/PA dual-mode imaging and PTT/chemo synergistic theranostic nanoparticles (DIST NPs). Experiment results showed that the DIST NPs had a long circulation *in vivo*, high bioavailability, high biocompatibility, and low effective dose. Thus, the DIST NPs showed an excellent NIFI/PAI dual-mode imaging and significant synergistic antitumor effect in hepatic carcinoma models.

### Lung Cancer

Chen et al. proposed to utilize habitat imaging-based ^18^F-fluorodeoxyglucose (^18^F-FDG) PET/CT radiomics for preoperatively discriminating non-small cell lung cancer (NSCLC) and benign inflammatory diseases (BIDs). Their study included 317 ^18^F-FDG PET/CT scans from patients who underwent aspiration biopsy or surgical resection. They constructed radiomics models based on clustering-based habitat radiomics method, conventional habitat-based method, and nonhabitat method. Experimental results showed that their adaptive habitat imaging-based method showed significantly improved discrimination performance compared to the conventional methods. This study implied that the microenvironmental variations in NSCLC and BID could be captured by PET/CT.

### Tumor Vessels

Zhou et al. utilized swept-source optical coherence tomographic angiography (SS-OCTA) to describe the morphologic characteristics of tumor-related vasculatures and their association with secondary choroidal neovascularization (CNV), subretinal fluid, choroidal thickness, and tumor decalcification in eyes with choroidal osteoma (CO), etc. Indocyanine green angiography identified inhomogeneous hyperfluorescence due to tumor-related vasculature, and all corresponded to the structures that appeared as sea-fan vascular networks combined with clusters of tangled vessels on SS-OCTA images. The identification of actual tumor vasculature in patients may help facilitate understanding of their pathogenesis, tumor control, and response to treatment.

## Imaging Methods for Cancer Research

### BLT Imaging

Yu et al. proposed a deep-learning optical reconstruction method based on one-dimensional convolutional neural networks (1DCNN) to improve the reconstruction accuracy of positioning and reconstruction efficiency of bioluminescent tomography (BLT). Compared with the reconstruction method based on multilayer perceptron, the training parameters in the 1DCNN were reduced and the learning efficiency of the model was improved. They used simulations to validate the superiority and stability of the 1DCNN method, and implemented animal experiments to further show the potential of the proposed method in practical applications.

Liu et al. presented a new multispectral difference strategy (MDS) to improve the accuracy of the BLT reconstruction based on analyzing the errors generated from the simplification from radiative transfer equation to diffusion approximation and data acquisition of the imaging system. The forward simulations showed that MDS can reduce the systematic errors in the process of light transmission. The inverse simulations and *in vivo* experiments showed that MDS was able to alleviate the ill-posedness of the inverse problem of BLT. The experiment results demonstrated that the MDS method had superior location accuracy, morphology recovery capability, and image contrast capability.

### CLT Imaging

Wang et al. proposed a prior compensation algorithm to carry out Cerenkov luminescence tomography (CLT) reconstruction based on depth calibration strategy. Since the attenuation of light in the tissue depends heavily on the depth, a depth calibration matrix was designed to calibrate the attenuation between the surface light flux and the density of the internal light source. The feature of the proposed algorithm was that the depth calibration matrix directly acts on the system matrix, rather than modifying the regularization penalty items. The experiment results showed that the proposed method could locate the radiation sources accurately by using single-view measurements.

Wei et al. proposed a probabilistic energy distribution density region scaling (P-EDDRS) framework to implement CLT reconstruction. In this framework, multiple reconstruction iterations were performed, and the Cerenkov source distribution of each reconstruction was treated as random variables. The size of the region required for the next operation was determined dynamically by combining intensity characteristics. Besides, each reconstruction source distribution was given a probability weight value. Experimental results showed that this reconstruction framework had better positioning accuracy and shape recovery ability.

### DOT Imaging

Wang et al. proposed a data self-calibration method based on a high-density (HD) parallel-plate diffuse optical tomography (DOT) system. Based on their proposed scheme, the reference predicted data can be constructed directly from the measurement data with the support of the HD-DOT system, which has nearly a hundred sets of measurements at each SD distance. The proposed scheme had been validated by Monte Carlo simulation, breast-size phantom experiments, and clinical trials, and results showed that the scheme had feasibility in ensuring the quality of the DOT reconstruction while effectively reducing the complexity.

### XLCT Imaging

Liu et al. proposed a new finite element mesh regrouping strategy-based hybrid light transport model for X-ray luminescence computed tomography (XLCT). In their scheme, two separate meshes were obtained and the system matrixes and source weight matrixes were separately calculated. Then, the two system matrixes with different dimensions were coupled, and the two meshes were combined to establish the hybrid optical transmission model. The proposed method can reduce the computational memory consumption significantly, thus achieving a good balance between computational accuracy and efficiency.

### Pathology Imaging

Zeng et al. proposed a wide-field pixel super-resolution color lensfree microscopy by performing wavelength scanning pixel super-resolution and phase retrieval simultaneously on the three channels of red, green, and blue (RGB), respectively. A high-resolution RGB three-channel composite color image is converted to the YUV space for separating the color component and the brightness component, keeping the brightness component unchanged as well as enhancing the color component through an average filter. The proposed method can eliminate the common rainbow artifacts of holographic color reconstruction and maintain the high-resolution details under different color illuminations.

## Reviews for Optical Imaging

Wang et al. reviewed recent advances in hybrid light propagation models, with particular emphasis on their powerful use for 3D optical imaging in cancer detection. Since traditional optical imaging can only qualitatively detect 2D biomedical information, 3D imaging technology is explored to provide 3D quantitative information. For 3D imaging, the light propagation models that reflect the interaction between light and biological tissues are important bases and extensively reviewed in this paper.

## Author Contributions

GZ wrote the text. All others were co-editors of the Research Topic and edited the text. All authors contributed to the article and approved the submitted version.

## Funding

This work was partially supported by the National Key Research and Development Program of China (No. 2017YFA0700401), the National Natural Science Foundation of China (No. 61871022), the Beijing Natural Science Foundation (No. 7202102), and the 111 Project (No. B13003).

## Conflict of Interest

The authors declare that the research was conducted in the absence of any commercial or financial relationships that could be construed as a potential conflict of interest.

## Publisher’s Note

All claims expressed in this article are solely those of the authors and do not necessarily represent those of their affiliated organizations, or those of the publisher, the editors and the reviewers. Any product that may be evaluated in this article, or claim that may be made by its manufacturer, is not guaranteed or endorsed by the publisher.
